# Recent Advancements in the Generation and Application of Therapeutic Cell Populations for Lung Epithelial Repair

**DOI:** 10.1155/term/8367426

**Published:** 2025-12-30

**Authors:** Muyang Zhou, Dana Brinson, Cindy Lei, Golnaz Karoubi

**Affiliations:** ^1^ Latner Thoracic Research Laboratories, University Health Network, Toronto, Ontario, Canada, uhn.ca; ^2^ Department of Laboratory Medicine and Pathobiology, Temerty Faculty of Medicine, University of Toronto, Toronto, Ontario, Canada, utoronto.ca; ^3^ Institute of Biomedical Engineering, University of Toronto, Toronto, Ontario, Canada, utoronto.ca; ^4^ Department of Molecular Genetics, Temerty Faculty of Medicine, University of Toronto, Toronto, Ontario, Canada, utoronto.ca; ^5^ Department of Mechanical and Industrial Engineering, University of Toronto, Toronto, Ontario, Canada, utoronto.ca

**Keywords:** cell therapy, directed differentiation, disease modeling, lung epithelial regeneration, pluripotent stem cells, respiratory diseases

## Abstract

Chronic respiratory diseases are a major global health concern. Lung epithelial dysfunction is a common underlying feature of many such conditions; hence, reconstructing the diseased epithelium with functional epithelial cells is a promising therapeutic approach. There are various endogenous stem cell and progenitor populations in the lung epithelium that can be utilized for transplantation. Additionally, pluripotent stem cells (PSCs) have emerged as a valuable source for generating therapeutic cells due to their capacity for indefinite self‐renewal and the availability of directed differentiation protocols to transform them into lung progenitors or mature lung epithelial cells. This review discusses the endogenous stem cell and progenitor populations of the lung epithelium, recent advances in developing directed differentiation protocols to generate these cells, and the application of both endogenous and PSC‐derived lung epithelial cells for disease modeling in vitro and as cell therapies in vivo. It provides valuable insights into the current progress of regenerative medicine within the respiratory field and highlights areas that require further research.

## 1. Introduction

Chronic respiratory diseases, such as chronic obstructive pulmonary disease (COPD), interstitial lung disease (ILD), cystic fibrosis, and pneumoconiosis, are among the most prevalent noncommunicable diseases worldwide [1]. Many of these conditions have a poor prognosis and will inevitably progress to an end stage, leading to lung failure. Existing therapies are mostly targeted toward alleviating symptoms, with none offering a curative solution. It is widely acknowledged that lung epithelial dysfunction is an integral and shared component across many chronic respiratory diseases. Therefore, cell therapy aiming at reconstructing the diseased lung epithelium with normal, functional cells has emerged as a promising approach in recent years.

Various cell populations can facilitate the repair and regeneration of lung tissue and thus have been considered for cell therapy. Multipotent bone marrow–derived cells (BMCs) attracted significant interest at the start of the century, largely fueled by several studies that reported the presence of intravenously infused BMCs in the lungs of recipient animals [2, 3]. However, their true engraftment and functional efficacy have been questioned [4, 5]. The recent trend in the field has shifted toward utilizing endogenous epithelial cells in the lung, as well as pluripotent stem cell (PSC)–derived lung epithelial cells. PSCs can serve as an inexhaustible source of therapeutic cells, making them an ideal candidate for lung cell therapy. Studies have established protocols for the direct differentiation of PSCs into lung progenitors, as well as mature cell types lining the airway and alveolar epithelium. These protocols have been applied to generate more clinically relevant and personalized disease models consisting of lung epithelial cells differentiated from patient‐derived PSCs, which has greatly benefited research into disease mechanisms and drug screening processes. More recently, endogenous and PSC‐derived lung epithelial cells have been transplanted into animal models as cell therapies to evaluate their functionality and therapeutic efficacy in vivo.

We review the cell populations that can be leveraged for lung epithelial repair and regeneration, recent advances in the directed differentiation of PSCs to derive those cells, their in vitro use for disease modeling, and in vivo application as cell therapies.

## 2. Endogenous Stem Cells and Progenitor Populations in the Lung Epithelium

**Figure 1:**
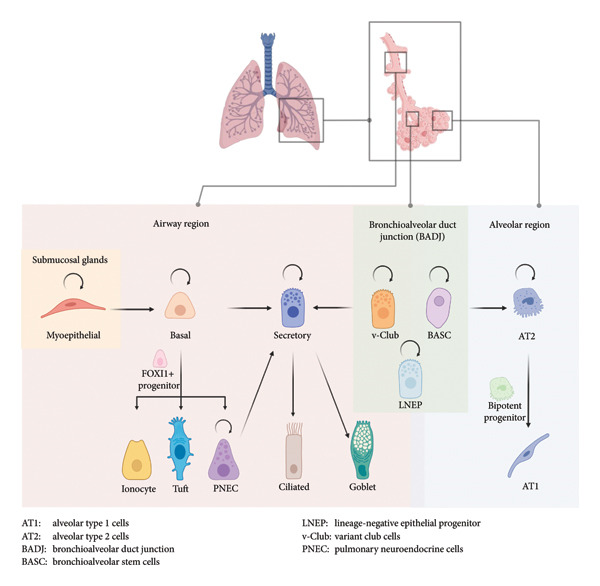
Summary of stem cell and progenitor populations in the lung epithelium. Curved arrows indicate the ability to self‐renew. Straight arrows indicate the ability to give rise to another cell type. Created with Biorender.com.

The airway and alveolar regions of the mammalian lung are lined by different types of epithelial cells on their luminal surface. The trachea through the bronchi is lined by a layer of pseudostratified epithelium composed mainly of basal cells, ciliated cells, and nonciliated secretory cells including goblet, serous, and club cells. Rare populations, such as chemosensory tuft cells, neuroendocrine cells, and ionocytes, are also present [6]. Along the proximal–distal axis of the airway, the epithelium gradually transitions from pseudostratified to simple cuboidal, accompanied by changes in its cellular composition. The frequencies of basal, ciliated, and goblet cells progressively decrease, whereas club cells become predominant [7]. The alveolar epithelium comprises type I (AT1) cells for gas exchange and type II (AT2) cells responsible for surfactant secretion, coordinating innate immune responses and maintaining alveolar homeostasis. Among the various cell types, basal cells and AT2 cells, given their capacity for self‐renewal and multilineage differentiation, are considered the endogenous stem cells of the lung epithelium. However, several facultative progenitor populations also exist. Though normally quiescent, they can proliferate and differentiate to replenish specific cell types following injury (Figure 1).

### 2.1. Airway Basal Cells

In rodents, basal cells are largely restricted to the trachea and main bronchi, whereas in humans, the pseudostratified epithelium containing basal cells extends distally to the terminal bronchioles [8]. In vivo lineage tracing in mice showed that basal cells are capable of long‐term self‐renewal and differentiation into club and ciliated cells during postnatal growth, in adult lungs at steady state, and in response to injuries [9–11]. Moreover, evidence strongly suggests that basal cells first differentiate into club cells, which then give rise to ciliated cells [6, 12]. Single‐cell RNA sequencing (scRNA‐seq) analysis identified a unique cytokeratin (KRT)4^+^/KRT13^+^ population in mouse trachea that may represent a transitional state between basal and club cells [6, 13]. Xenograft models demonstrated that basal cells could repopulate epithelium‐denuded rat trachea and restore a fully differentiated, functional airway epithelium composed of basal, secretory, and ciliated cells [14, 15]. In vitro, basal cells isolated from mouse trachea and human bronchi self‐renew, differentiate, and spontaneously organize to form “tracheospheres” containing secretory and ciliated cells in the absence of other epithelial or stromal populations [9]. Basal cells are also the predominant source of tuft cells, neuroendocrine cells, and ionocytes [6]. Therefore, for diseases that affect the airways, such as cystic fibrosis and primary ciliary dyskinesia, transplantation of healthy basal cells is a viable strategy to reconstitute the epithelium with functional airway cells.

### 2.2. Secretory Club Cells

Ciliated cells have been shown to be terminally differentiated [16]. Among the secretory cell cohort, there is no evidence of goblet or serous cells demonstrating any regenerative capacity. In contrast, Scgb1a1 lineage‐labeled club cells, which constitute the majority of secretory cells in the distal airways, undergo self‐renewal and generate ciliated cells during postnatal growth, in steady‐state adult lungs and following epithelial injury [12, 17]. Club cells in the proximal airways contribute minimally to epithelial regeneration at steady state but can give rise to goblet cells when stimulated by antigens such as ovalbumin [18]. Club cells can also dedifferentiate into basal cells in vivo when triggered by the loss or ablation of native basal cells and ex vivo in the absence of basal cells [12, 19]. These dedifferentiated cells showed multilineage differentiation potential and functioned as well as their endogenous counterparts in repairing epithelial injury [19].

### 2.3. Variant Club (v‐Club) Cells

Studies have identified a population of secretory cell‐like airway progenitors, termed variant club (v‐Club) cells, that have low expression of club cell secretory protein (CCSP; CC10; Scgb1a1) but are enriched in secretoglobin family 3A member 2 (Scgb3a2) and uroplakin 3a [20, 21]. Unlike canonical club cells, v‐Club cells are resistant to naphthalene injury, presumably due to their lack of the cytochrome P450 2F2 isoenzyme, which converts naphthalene into its cytotoxic metabolites [22]. v‐Club cells are in close proximity to neuroepithelial bodies, which are primarily found at airway bifurcations and at the bronchioalveolar duct junction (BADJ) [20, 21, 23, 24]. v‐Club cells proliferate after naphthalene treatment and give rise to club and ciliated cells, and those at the BADJ can also contribute to alveolar repair in response to bleomycin by generating AT1 and AT2 cells [21, 25]. These findings support the existence of spatially distinct progenitor niches in the airways that maintain epithelial integrity and diversity after injury.

### 2.4. Bronchioalveolar Stem Cells (BASCs)

The BADJ is home to another population of facultative progenitors that coexpress CCSP and surfactant protein C (SPC), termed BASCs [26, 27]. Similar to v‐Club cells, BASCs express low levels of cytochrome P450 2F2, potentially accounting for their resistance to naphthalene‐induced injury and other xenobiotics [27]. BASCs proliferate in response to bronchiolar and alveolar injury and display clonogenicity, self‐renewal, and multilineage differentiation capacity in vitro [26]. Recent lineage‐tracing experiments have confirmed the existence of BASCs as a distinct population in vivo and demonstrated their ability to generate club cells, ciliated cells, and AT1 and AT2 cells in murine lung following different types of epithelial injury [27, 28]. It is important to note that the BADJ is absent in human airways. Therefore, the existence of an equivalent facultative progenitor population in humans remains uncertain.

### 2.5. Other Airway Progenitor Cells

The increasing availability of scRNA‐seq data and animal models with reporter systems for lineage tracing has greatly expanded our understanding of rare airway cell types. Several groups observed that pulmonary neuroendocrine cells (PNECs) become proliferative after naphthalene injury or selective ablation of club cells and give rise to club and ciliated cells [29–32]. Ouadah et al. argue that only a small subset of PNECs is proliferative and capable of migrating toward the injured region [33]. scRNA‐seq analysis identified a forkhead box I1 (FOXI1) lineage‐labeled progenitor population of PNECs, tuft cells, and ionocytes that coexpress markers of all three cell types [34]. Myoepithelial cells of the submucosal glands transiently expand and migrate to the surface airway epithelium where they assume a basal cell–like phenotype following severe injury to facilitate repair [35, 36]. These cells demonstrate multipotency and contribute to the regeneration of club, ciliated, and goblet cells, as well as glandular cell types [35, 36]. Although these rare populations may be of lesser priority for cell therapies, they help delineate intracellular signaling pathways, such as WNT [37] and Notch [13, 38], that promote proliferation, maintenance of stem cell properties, or bias cell fate to regenerate specific epithelial populations.

### 2.6. Alveolar Type 2 (AT2) Cells

As early as the 1970s, researchers reported that AT2 cells can function as progenitors of AT1 cells. During postnatal development, AT2 cells exhibit robust proliferation, traverse through an intermediate state that shows characteristics of both AT1 and AT2 cells, and subsequently transdifferentiate into AT1 cells [39, 40]. More recently, several groups showed that AT1 and AT2 cells arise through maturation of a bipotent progenitor population during the saccular stage of pulmonary development [41]. In adult lungs at steady state, AT2 cells self‐renew at a slow rate and only occasionally transdifferentiate into AT1 cells, primarily at discrete foci concentrated in the perivascular and peripheral regions of the lung [41, 42]. AT2 cells become activated and rapidly regenerate AT1 cells to repair the alveolar epithelium in response to hyperoxia, which selectively damages AT1 cells and bleomycin‐induced pulmonary fibrosis [41, 43]. Following AT2‐specific alveolar injury, these cells undergo robust clonal expansion to replenish the AT2 compartment, as well as infrequent differentiation to AT1 cells [42]. These findings suggest that AT2 cells exhibit phenotypic plasticity, enabling them to bias cell fate toward either alveolar cell type in response to different types of injuries. Additionally, lineage‐labeled AT2 cells cultured under 3D conditions spontaneously form alveolospheres that contain both AT1 and AT2 cells [42].

### 2.7. Alveolar Type 1 (AT1) Cells

Conversely, AT1 cells are generally considered to be terminally differentiated. Lineage‐labeled Hopx^+^ AT1 cells self‐renew and very rarely give rise to Spc^+^ AT2 cells during compensatory regrowth of the adult murine lung following pneumonectomy and in 3D organoid cultures [44]. Nevertheless, it remains uncertain whether this Hopx/Spc coexpressing population represents truly bipotent AT1 cells or is merely an intermediate state that AT2 cells traverse through to regenerate AT1 cells as described by previous studies [39, 40]. Another study reported that ectopic expression of SOX2, a transcription factor that is normally restricted to the airways and promotes airway differentiation, can reprogram terminally differentiated AT1 cells to become proliferative, downregulate AT1 markers, and express the basal cell marker p63, suggesting that AT1 cells possess some degree of phenotypic plasticity [45].

Although there has been some success with the isolation and culture of primary epithelial cells, the process remains largely invasive, and it is difficult to maintain their function and phenotype in vitro. PSCs offer a promising alternative for developing cell therapies, as they eliminate the need for invasive procedures and are capable of indefinite self‐renewal when maintained in a pluripotent state. Directed differentiation protocols enable the generation of various lung epithelial cell types, complemented by sorting strategies to purify populations for transplantation. In recent years, significant progress has been made in refining these differentiation protocols.

## 3. Advances in Lung‐Directed Differentiation Protocols

**Figure 2:**
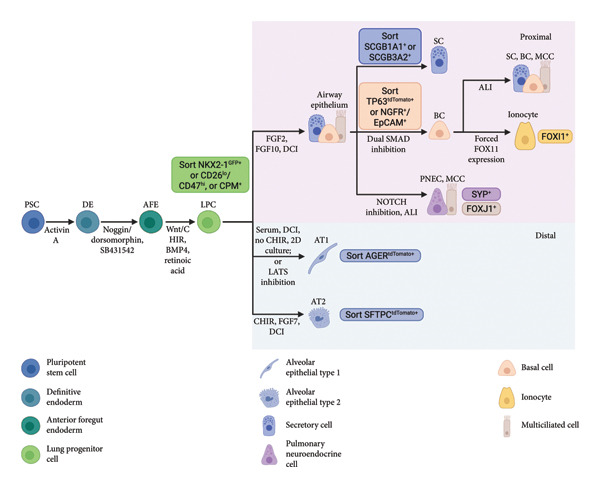
Summary of directed differentiation protocols to differentiate pluripotent stem cells (PSCs) into lung epithelial populations. Created with Biorender.com.

Generally, protocols for the directed differentiation of PSCs into lung epithelial lineages aim to mimic the fetal lung developmental trajectory. The lung originates from the definitive endoderm (DE) layer and emerges as two buds from the ventral–lateral wall of the anterior foregut region [46]. The two buds then undergo a repeated cycle of elongation into the surrounding mesoderm and branching morphogenesis, ultimately forming the respiratory tree [47]. This process is directed by a series of spatially and temporally regulated morphogenic factors, many of which have become the basis for differentiation protocols (Figure 2).

It was first established that activin A can induce DE formation from embryonic stem cells (ESCs) [48–51]. DE cells emerge from a brachyury‐expressing population initially with both mesoderm and endoderm potentials. Activin A, which mimics high levels of Nodal signaling during gastrulation [52], favors an endodermal program while decreasing the expression of mesoderm markers. Isolation of cells expressing DE markers can further enrich DE cells to near homogeneity [48, 51]. Of note, CXCR4 is a useful marker for distinguishing DE cells from contaminating visceral endoderm cells and undifferentiated ESCs [50].

Studies have attempted to generate lung epithelial cells directly from the endoderm stage, but the efficiency is low due to the presence of mixed cell populations [53–56]. Cell fate can be better established by generating progressively lineage‐restricted progenitors. Thus, differentiating PSCs toward an anterior foregut fate first is advantageous. Activin A–induced DE can spontaneously differentiate into both anterior and posterior phenotypes [57]. DE cells fated to become anterior foregut endoderm (AFE) are exposed to the Nodal antagonist Lefty and the bone morphogenic protein (BMP) antagonist Cerberus in vivo, suggesting that inhibiting transforming growth factor (TGF)‐β1 superfamily signaling is necessary for AFE specification [58, 59]. Indeed, the combination of NOGGIN and SB‐431542, inhibitors of BMP and TGF‐β signaling, respectively, specifies an AFE phenotype in DE cells [57]. Replacing Noggin with another BMP inhibitor, dorsomorphin, generates similar outcomes [60]. Exposure to fibroblast growth factor 2 (FGF2) and sonic hedgehog (SHH), factors involved in branching morphogenesis, also results in upregulation of anterior foregut transcription factors SOX2 and NKX2.1 [61].

The lung buds emerge from the ventral side of the AFE in response to WNT, BMP, and FGF signals from the surrounding mesoderm [62]. In particular, the combination of WNT3a, KGF (FGF7), FGF10, BMP4, and EGF (collectively, WKFBE) induces the expression of a key transcriptional regulator of lung development, NKX2.1 (thyroid transcription factor 1 [TTF1]), in AFE cells [57, 63–65]. The NKX2.1^+^ population uniquely upregulates several lung epithelial markers, including SPC, CCSP, p63, and FOXJ1, when cultured in WKFBE ventralization medium [63]. Consequently, protocols commonly use fluorescent reporter systems to isolate the NKX2.1^+^ population. Sorting strategies based on cell surface proteins, such as CD47^hi^/CD26^-^ [66, 67] and carboxypeptidase M (CPM)^+^ [67, 68], can also achieve the same goal. For human PSC differentiation, WNT3a is replaced by CHIR99021, a small molecule that mimics WNT signaling [60]. It was later discovered that WNT3a and BMP4 alone are sufficient to specify lung progenitors from AFE cells [69]. Similarly, CHIR99021, BMP4, and RA (collectively, CBRa) are sufficient for deriving NKX2.1+ progenitors from human iPSCs [68, 70, 71].

The growth factors FGF2 and FGF10 are necessary for inducing the expression of lung epithelial genes [63, 64] and maintaining the proliferation of lung epithelial progenitors [72]. Exposing fetal lung epithelial cells to cyclic adenosine monophosphate (cAMP) and steroid‐containing medium promotes surfactant protein production, indicative of functional AT2 cells [73]. Addition of dexamethasone, cAMP, isobutylmethylxanthine (IBMX), and KGF (collectively, DCI + K) to the differentiation medium resulted in further augmentation of lung epithelial marker expression [63]. The combination of FGF2 + FGF10 followed by DCI + K conditioning has been proven to be widely applicable in various PSC differentiation protocols [66, 70, 74–76]. Activation of the WNT signaling pathway in NKX2.1+ lung progenitors promotes a distal lung phenotype, whereas withdrawal of WNT patterns lung progenitors into proximal airway lineages [75]. This finding became crucial for differentiating PSCs into proximal and distal lung epithelial progenitors and their derivatives, such as airway basal cells [76] and AT2 cells [70]. Lastly, 3D culture conditions allow lung progenitor cells to self‐organize into monolayered epithelial organoids resembling primary lung epithelium [69, 77, 78]. The organoids showed higher expression of epithelial genes and reduced mesenchymal gene expression compared to 2D cultures [67, 69]. Thus, 3D conditions have been widely adopted, mostly using highly defined growth factor–reduced Matrigel for better control over the differentiation process and resulting cell lineages.

Recent advancements have focused on minor modifications to facilitate the differentiation and proliferation of specific cell types, such as the addition of small molecules and the development of specialized media. For example, dual SMAD inhibition by A83‐01 and DMH‐1 promotes the long‐term expansion of airway basal cells while maintaining their capacity to differentiate into functional airway epithelia [79]. Studies have successfully derived basal cells from PSC‐derived airway organoids by culturing in an optimized medium containing A83‐01 and DMH‐1 [76, 80, 81]. Forced expression of the transcription factor FOXI1 can further specify ionocyte progenitors from basal cells [13, 82]. Engineered mouse and human PSC lines carrying reporter systems targeted to secretoglobin family members Scgb1a1 and Scgb3a2 enable the labeling, purification, and profiling of airway secretory cells [83]. PNECs can also be enriched by air–liquid interface (ALI) culture conditions and inhibition of NOTCH signaling [84, 85]. Studies recapitulated the endogenous sequence of AT2 development and sequentially sorted for NKX2.1+ lung progenitors followed by NKX2.1+/SFTPC+ distal tip‐like cells to purify AT2 cells [70, 86]. For mouse ESC differentiation, Herriges et al. modified the ventralization medium to mimic the switch from RA to FGF signaling soon after NKX2.1 induction [87], which resulted in a significant increase in the frequency of NKX2.1+ cells [88]. Culturing NKX2.1+ cells in a specialized lung progenitor medium [89] facilitated the generation of distal lung tip‐like progenitors, which assumed the molecular phenotypes of AT1 and AT2 cells in vivo [88]. AT1 cells are typically absent from alveolar organoids, but transferring PSC‐derived distal lung tip‐like cells to 2D culture enables them to rapidly adopt a squamous morphology and upregulate AT1 markers [70]. Recent work discovered that nuclear YAP signaling promotes a transcriptomic shift from AT2 to AT1 cell fate, and the resulting cells resemble native AT1 cells at the molecular, morphological, and functional levels [90]. This led to the development of a differentiation medium containing a large tumor suppressor kinase (LATS) inhibitor, which facilitates nuclear localization of YAP [91], that can efficiently differentiate PSC‐derived AT2 cells into AT1 cells. Removal of WNT, FGF, and TGF‐β signaling can also direct airway epithelial progenitors toward an AT2 cell fate, whereas activation of BMP signaling promotes differentiation into AT1 cells [92].

Although significant progress has been made in the development of directed differentiation protocols, there remains work to be done in identifying the conditions that promote cell maturation to fully replicate the adult lung phenotype. In vivo implantation of PSC‐derived lung progenitors may promote their maturation into functional epithelial cells [77, 80, 88]. In vitro culture systems also influence cell fate. For example, ALI cultures promote differentiation of functional airway cell types that are not seen in 3D organoids [61, 93], as well as maturation of induced AT2 cells [94]. Coculturing with lung‐specific mesenchyme can also increase the yield of NKX2.1+ lung progenitors and influence their differentiation program [95].

The applications of endogenous and PSC‐derived lung epithelial cells can be broadly classified into two categories: in vitro for disease modeling and in vivo as cell therapies. PSCs provide an indefinitely renewable source of human cells, thereby enabling the development of models that faithfully capture patient‐specific disease characteristics and are well‐suited for high‐throughput drug screening. Therefore, our discussion of in vitro applications will focus on these PSC‐derived models of respiratory diseases. Conversely, the in vivo application of endogenous and PSC‐derived lung epithelial cells is a relatively recent endeavor. Encouragingly, numerous studies have demonstrated the safety and efficacy of transplanting these cells into small animal models for treating various respiratory diseases. The remainder of this review will discuss both in vitro and in vivo applications, emphasizing recent advancements in *in vivo* studies and key practical considerations that could accelerate the clinical translation of these cells into viable cell therapies.

### 3.1. In Vitro Applications of PSC‐Derived Models to Study Lung Diseases

#### 3.1.1. Induction of Disease in Healthy PSC‐Derived Models

Models derived from healthy PSCs can be induced into a diseased state using pathogenic agents or methods that simulate disease processes in humans or animals. Idiopathic pulmonary fibrosis (IPF) is a commonly modeled lung disease. Bleomycin, a fibrotic agent used to induce IPF in mouse models, can trigger a fibrotic phenotype in human iPSC‐derived alveolar organoids [96]. Gene ontology analysis revealed an enrichment of pathways associated with fibroblast activation, cellular senescence, and extracellular matrix (ECM) accumulation compared to DMSO‐treated organoids. Unlike bleomycin‐induced IPF in mouse models, which resolves rapidly, treated organoids tend to maintain their diseased state [96, 97]. TGF‐β1, a key mediator of tissue fibrosis, promotes fibroblast‐to‐myofibroblast transition and cell contraction, leading to scarring in lung organoids that resemble fibroblastic foci seen in IPF [98]. Similarly, iPSC‐derived AT2‐like cells incubated with cytokine cocktails containing TGF‐β1, tumor necrosis factor (TNF)‐α, and interleukin (IL)‐1β, all of which are linked to IPF pathogenesis, exhibited upregulation of profibrotic factors and IPF‐associated transcriptomic changes [99, 100]. CRISPR‐Cas9‐mediated deletion of Hermansky–Pudlak syndrome 1 (HPS1) can also promote fibrotic changes including ECM accumulation and mesenchymal cell expansion in PSC‐derived lung bud organoids [77, 92].

PSC‐derived models are also an excellent tool to study infectious lung diseases, particularly viral infections. Various strains of influenza viruses can replicate productively in PSC‐derived airway organoids, with replication kinetics resembling those observed in ex vivo human bronchus explants [101, 102]. Zhou et al. developed a 2D airway monolayer from the airway organoids that provided better access to the apical surface during viral inoculation [103]. As such, these models are valuable for studying the tropism and infectivity of influenza viruses. Due to the pandemic, PSC‐derived organoid models have been adapted to study the biology and pathogenesis of SARS‐CoV‐2 infections [104–108]. Studies have shown that SARS‐CoV‐2 can readily infect these organoids, primarily targeting PSC‐derived AT2‐like cells [104–106]. Transcriptomic analysis of these organoids revealed a shift toward an inflammatory phenotype, marked by a robust induction of Type I and III interferon pathways [107], chemokine production [108], and the loss of mature alveolar markers [104]. Other respiratory viruses, such as respiratory syncytial virus, measles, and parainfluenza virus, have also been faithfully modeled using human stem cell–derived lung organoid models [77, 109].

#### 3.1.2. Generation of Disease Models From Patient‐Derived PSCs

Patient‐derived PSCs offer an alternative cell source for establishing respiratory disease models, particularly for those associated with genetic mutations that are otherwise difficult to model with exogenous methods. These cells enable the exploration of how patient‐specific mutations contribute to disease development, as well as the screening of medications and therapies that are particularly effective for the individual or specific patient groups.

Cystic fibrosis is one of the commonly modeled diseases using patient‐derived PSCs. It was first established that CFTR‐expressing airway epithelial cells could be derived from human iPSCs by guiding them through an endodermal developmental pathway, followed by differentiation at an ALI [61, 110]. Similarly, patient‐derived iPSCs carrying the F508del mutation can be subjected to the same protocol, with resulting cells faithfully modeling the CFTR trafficking defect that is characteristic of F508del CF patients. Subsequent studies demonstrated that airway organoids derived from CF patient iPSCs and bronchoalveolar lavage cells can recapitulate functional abnormalities, such as impaired forskolin‐induced ion flux and the presence of a thick mucus layer on the apical surface [75, 76, 111]. The healthy endogenous CFTR locus in human PSCs may also be replaced with a diseased CFTR transgene lacking the F508 residue [112]. The development of these models has enabled the testing of CFTR‐correcting strategies, including zinc finger nuclease‐mediated [113] and CRISPR‐Cas9‐mediated homology‐directed repair [114] to introduce corrective sequences at the endogenous CFTR locus and CFTR modulators such as VX‐770 and VX‐809 to restore its expression and function [61, 111]. Berical et al. observed genotype‐specific differences in the response of patient iPSC‐derived airway organoids to CFTR modulators, suggesting that such models are effective at screening candidate therapeutics [115]. More recently, a high‐throughput platform using patient‐specific iPSC‐derived lung progenitor cells has enabled the modeling of both common and rare CF mutations, as well as the assessment of patient‐specific responses to therapeutic compounds, marking a significant step forward in personalized medicine [116]. In addition to cystic fibrosis, PSC‐derived organoid models have been employed to study other respiratory diseases linked to genetic mutations, including primary ciliary dyskinesia [76, 117], HPS [118], and ILD associated with surfactant protein gene mutations [70, 119].

Genome editing technologies are being increasingly applied to patient‐derived PSC models, either to introduce disease‐causing mutations or as corrective therapies. For example, CRISPR‐Cas9 has been used to introduce frameshift mutations in HPS1, HPS2, and HPS4 genes of human ESCs, all of which are implicated in HPS‐associated interstitial pneumonia [77, 120]. The lung organoids derived from these cells exhibited fibrotic phenotypes. CRISPR‐Cas9 knockdown of ADGRG6, a gene associated with COPD, reduced the inflammatory response of iPSC‐derived AT2 cells when exposed to cigarette smoke [121]. CRISPR‐based gene correction of SFTPB mutations restores surfactant production and processing in iPSC‐derived AT2 cells [70]. Small interfering (si)RNA targeting the SMG1 transcript in a CF patient‐derived iPSC model resulted in increased CFTR expression and function [115]. An ACE2‐null iPSC line generated via nonhomologous end joining inhibited the entry of SARS‐CoV‐2 viral particles compared to unedited iPSCs [122].

### 3.2. In Vivo Applications of Endogenous and PSC‐Derived Lung Epithelial Cells as Cell Therapies

In principle, endogenous stem cells, progenitors, and PSC‐derived epithelial populations can all be used for transplantation as cell therapies. Table 1 summarizes the various cell populations that have been applied in *in vivo* studies and their corresponding outcomes.

**Table 1:** Summary of in vivo applications of endogenous and PSC‐derived lung epithelial cells and their corresponding outcomes.

Cell source	Animal model	Route of administration	Outcomes and time point of evaluation	Reference
Airway stem/progenitor populations				
Primary mouse basal cells	Mouse	Subcutaneous	Formation of cystic structures with club and ciliated cells (8 months)	[79]
Primary mouse p63^+^Krt5^+^ basal cells	Mouse, influenza infection	Intratracheal	Differentiation into AT1, AT2, and club cells at sites of inflammation, reduced interstitial tissue damage (90 days)	[123]
Primary human SOX9^+^ basal cells	Mouse, bleomycin‐induced fibrosis	Intratracheal	Large‐scale incorporation into distal lung and alveolar regions, reduced fibrosis, improved pulmonary function (3 weeks)	[124]
Primary mouse SOX9^GFP+^ tdTomato^+^ basal cells	Mouse, naphthalene, and bleomycin‐induced fibrosis	Intratracheal	Cell engraftment at the site of injury and region‐specific differentiation: differentiation into all major airway epithelial cell types in naphthalene‐injured lungs and differentiation in AT1 and AT2 cells in bleomycin‐injured lungs, comprising ∼2%–10% of lung epithelium (4 and 8 weeks)	[89]
Primary human fetal basal cells	Rat, bleomycin‐induced fibrosis	Intratracheal	Reduced fibrosis, improved alveolar structures, downregulation of profibrotic factor TGF‐β (28 days)	[125]
Human iPSC‐derived NKX2.1^GFP^TP63^tdTomato^ basal cells	Rat, decellularized tracheal xenograft	Subcutaneous	Formation of pseudostratified, differentiated airway epithelium similar to in vivo native airways (3 weeks)	[76]
Mouse ESC‐derived NK2.1^mCherry+^ NGFR^+^GFP^+^ basal cells	Mouse, tracheal denudation by polidocanol	Intratracheal	Reconstitution of tracheal epithelium, long‐term self‐renewal, and multilineage differentiation into other airway cell types (3 weeks up to 2 years)	[80, 81]
Mouse ESC‐derived NK2.1^mCherry+^ NGFR^+^GFP^+^ basal cells	Mouse, polidocanol‐induced intrapulmonary airway denudation via microbronchoscopy	Intrapulmonary	Engraftment of ESC‐derived basal cells into intrapulmonary airways and differentiation into secretory and ciliated cells (days 2, 10, and 42)	[142]
Human iPSC‐derived airway progenitors on silk fibroin‐collagen vitrigel membrane	Pig, removal of tracheal segment	Ex vivo tracheal repair using patch	Formed native‐like mucociliary epithelium containing ciliated and goblet cells (3 days)	[157]
Rare/Niche airway progenitor populations				
Primary mouse myoepithelial cell	Mouse, decellularized tracheal graft	N/A (ex vivo)	Myoepithelial cell‐derived basal‐like cells engraft, proliferate, and generate luminal epithelial cells to repopulate the denuded trachea (30 days)	[35, 36]
Primary mouse CC10^–^β4^+^ LNEPs	Mouse, influenza infection	Intranasal	Formation of clusters of AT2 cells surrounding native alveoli, as well as Krt5‐ and CC10‐expressing cells near endogenous airway cells (21 days)	[126]
Primary mouse β4+/H2‐K1^high^ club‐like cells	Mouse, bleomycin‐induced fibrosis	Intratracheal	Differentiate into AT1 and AT2 cells covering larger areas of alveolar walls, improved oxygen saturation (24 days)	[127]
Alveolar stem/progenitor populations				
Primary mouse AT2 cells	Mouse, bleomycin‐induced fibrosis	Intratracheal	AT2 cell engraftment, reduced fibrosis, and inflammation (21 days)	[128]
Primary mouse AT2 cells	Mouse, influenza infection, acid instillation, bleomycin‐induced fibrosis	Intranasal	AT2 cell engraftment, transdifferentiation into AT1 cells, reduced collagen deposition and inflammatory cell infiltration, increased blood–oxygen saturation (13 days postinfection, 14 days post–acid instillation, and 23 days postbleomycin)	[129]
Primary mouse AT2 cells, SCA1+, and SCA1−	Mouse, bleomycin‐induced fibrosis	Intratracheal	SCA1‐AT2 cells exclusively contribute to alveolar regeneration, and SCA1+ AT2 cells repopulate both airway and alveolar regions (early: 14–24; mid: 42–84; late: 112–119 days)	[130]
Primary human AT2 cells	Mouse, bleomycin‐induced fibrosis	Oral aspiration	AT2 cell engraftment, transdifferentiation into metaplastic KRT5+ basal‐like cells (18–22 days)	[131]
Mouse ESC‐derived tip‐like progenitor cells	Mouse, bleomycin‐induced fibrosis	Intratracheal	Durable cell engraftment and maturation in alveolar regions, forming AT1 and AT2 cells (6 weeks–6 months)	[88]
Human PSC‐derived bud tip progenitors	Mouse, naphthalene	Intratracheal	Engraftment in trachea and bronchi, differentiation into multiciliated, goblet, and secretory cells (6 weeks)	[141]
Others				
Primary mouse and human fetal and adult unfractionated lung cells	Mouse, naphthalene (or replace with cyclophosphamide), total body irradiation, bleomycin‐induced fibrosis	Intravenous	Donor‐derived patches containing airway and alveolar epithelial, mesenchymal, and endothelial cells occupying up to 30% of total lung area, improved lung compliance, resistance, and tissue damping, attenuated fibrosis (30 days up to 4 months for naphthalene, 2 months for bleomycin)	[132–136]
Undifferentiated mouse PSCs	Mouse, endotoxin	Intravenous	Structural and functional improvements to the injured lung; downregulated inflammatory response and improved pulmonary function (24 and 48 h)	[137]
Human PSC‐derived NKX2.1+/FOXA2+ lung progenitors	Mouse, N/A	Underneath kidney capsule	Macroscopic growths containing cystic and tubular structures, differentiation into airway and alveolar epithelial cells (6 months)	[60]
Mouse ESC‐derived NKX2.1+ lung progenitors	Mouse, decellularized lung scaffolds	Intratracheal	Repopulation of alveolar regions, a subset acquired a flattened AT1 morphology (10 days)	[63]
Human PSC‐derived lung progenitors	Rat, detergent‐based local deepithelialization	Intrabronchial	Cell engraftment, differentiation into alveolar epithelial cells and KRT5+ pods, contribution to lung injury repair (10 days)	[143]
Rat fetal lung progenitors	N/A	Implantation of cells suspended in Gelfoam sponge into lung tissue	Formation of alveolar‐like structures around endogenous alveoli, neovascularization surrounding implanted scaffold (60 days)	[155]
Mouse lung progenitors	N/A	Subcutaneous, cells suspended in gelatin‐based 3D microbubbles	Formation of alveolar‐like structures around endogenous alveoli, neovascularization surrounding implanted cells (28 days)	[156]

#### 3.2.1. Transplantation of Endogenous Lung Stem Cell and Progenitor Populations

Numerous studies have transplanted basal cells due to their capacity to generate all airway cell types. Basal cells injected subcutaneously into immunodeficient NOD‐scid IL2Rγ^null^ (NSG) mice formed cystic structures with secretory and ciliated cells, suggesting multilineage differentiation capacity ectopically, but the relevance of this ectopic model to lung repair is limited [79]. Basal cells delivered intratracheally into influenza‐infected recipients engrafted into the interstitial and alveolar regions up to 90 days post‐transplantation, gave rise to AT1, AT2, and secretory cells, and reduced interstitial scarring; however, the study did not report any associated functional improvements [123]. A rare population of SOX9+ basal cells showed large‐scale incorporation into the distal lung of bleomycin‐injured NSG mice, forming sac‐like structures expressing AT1 markers and improving pulmonary function [124]. Although functional recovery was encouraging, durability beyond the 3‐week observation period was not established. A similar study reported engraftment of SOX9+ lung progenitors at the site of injury up to 8 weeks along with region‐specific differentiation: In naphthalene‐injured lungs, SOX9+ progenitors contributed to all major airway epithelial cell types, whereas in bleomycin‐injured lungs, they differentiated into AT1 and AT2 cells [89]. FACS quantification revealed that engrafted cells comprised ∼2%–10% of the lung epithelium. Basal cells isolated from human fetal lung ameliorated fibrosis in bleomycin‐injured rats 28 days post‐transplantation by decreasing collagen deposition, restoring alveolar structures, and downregulating expression of the profibrotic factor TGF‐β, yet whether these effects reflect stable epithelial replacement or transient paracrine modulation remains uncertain [125]. Together, these findings support the use of basal cells as a cell therapy for airway and alveolar regeneration.

Rare and niche airway populations have also been utilized for transplantation. Myoepithelial cells isolated from submucosal glands of adult mice are able to fully repopulate denuded tracheal xenografts ex vivo and differentiate into KRT5+ basal and KRT8+ luminal cells, including secretory and ciliated lineages [35, 36]. CC10^–^β4^+^ lineage‐negative epithelial progenitors (LNEPs) isolated from adult mouse lungs, when administered intranasally to influenza‐infected recipients, formed clusters of AT2 cells surrounding native alveoli but expressed Krt5 and CC10 near endogenous basal and secretory cells [126]. However, engraftment was not quantified and functional benefits were not reported. Another population of β4^+^/H2‐K1^high^ LNEPs isolated from adult mouse airways gave rise to AT1 and AT2 cells lining alveolar walls and, despite sparse, unquantified engraftment, significantly improved oxygen saturation in bleomycin‐injured NSG recipients up to 24 days post‐transplantation [127].

In the alveoli, AT2 cells stand out as a promising candidate for cell therapy. Adult donor‐derived AT2 cells have been shown to engraft into pulmonary injury models, including bleomycin‐induced fibrosis and *Pseudomonas* infection, and maintain their fate while also transdifferentiating into AT1 cells, as shown by immunostaining [128, 129]. Functionally, AT2 cell transplantation reduced collagen deposition and inflammatory cell infiltration in bleomycin‐injured mice and improved blood–oxygen saturation in influenza‐infected mice [128, 129]. Functional benefits were only evaluated ∼20 days after the onset of disease, and their persistence beyond this period remains unclear. Lineage‐traced AT2 cells lacking stem cell antigen 1 (SCA1) expression exclusively regenerated alveolar epithelial cells in bleomycin‐injured mice, whereas SCA1+ AT2 cells repopulated the airways and expressed airway epithelial markers up to 119 days after transplantation, highlighting the phenotypic plasticity of AT2 cells [130]. However, AT2 transplantation carries the risk of maladaptive plasticity, an issue largely unaddressed in most studies. Human AT2 cells transplanted into fibrotic hosts transdifferentiated into KRT5+ basal cells, which was exacerbated by the presence of a profibrotic mesenchyme, with up to 80% of engrafted patches forming dysplastic basaloid structures [131].

Yair Reisner and colleagues have explored the transplantation of unfractionated lung cells from lineage‐labeled adult mice, which form chimeric donor‐derived patches occupying up to 30% of total lung area by 16 weeks post‐transplant, albeit requiring 16 million cells per mouse [132–136]. Engraftment was quantified by systematic analysis of serial lung sections, calculating the percentage of GFP+ area relative to total lung tissue using automated image processing with blinded validation. Donor‐derived patches contained epithelial, mesenchymal, and endothelial cells, and epithelial cells in these patches expressed markers of AT1, AT2, and club cells. Recipients demonstrated improved compliance, resistance, and tissue damping. Moreover, the group recently established that coadministration of donor‐derived hematopoietic cells with lung progenitors enables engraftment across major genetic barriers without the need for chronic immunosuppression [134].

#### 3.2.2. Transplantation of PSC‐Derived Lung Epithelial Cells

Although cell therapies utilizing adult lung progenitors show promise, challenges such as their slow turnover rate, difficulty of procurement, and immunogenicity limit their widespread use. PSCs, with their capability for indefinite self‐renewal and multilineage differentiation, may provide an alternative solution. Genetic engineering techniques can also correct defects in cells and reduce tumorigenicity and immune rejection. Several studies have transplanted undifferentiated ESCs and iPSCs into mouse models of acute lung injury and noted improved histological features, lung injury scores, neutrophil accumulation, and proinflammatory cytokine production [137–139]. Nevertheless, the therapeutic benefits appear to be conferred by paracrine factors, as conditioned medium achieved similar effects [138, 139]. Cell engraftment was also shown to be minimal with local teratoma formation and cellular infiltration into other organs [138], highlighting the need for more differentiated populations. PSC‐derived endodermal cells injected underneath the kidney capsule of NSG mice persisted up to 5 weeks and differentiated exclusively to endodermal lineages, confirming their short‐term safety and phenotypic stability [48, 49, 51, 57, 140]. Human PSC‐derived NKX2.1+/FOXA2+ lung progenitor cells transplanted underneath the kidney capsule exclusively gave rise to lung epithelial cells expressing airway and alveolar markers after 6 months, further emphasizing the importance of transplanting lung‐fated populations [60]. NKX2.1+ lung progenitors can also repopulate decellularized mouse lung scaffolds [63]. 71% of cells maintained NKX2.1 expression, whereas some acquired a flattened morphology expressing the AT1 cell marker T1a. Human PSC‐derived bud tip progenitors engrafted in the trachea and bronchi of naphthalene‐injured mice, retained SOX2 expression but reduced SOX9 expression, and differentiated into multiciliated, goblet, and secretory cells 6 weeks post‐transplantation [141]. Engraftment, measured as the number of GFP^+^ patches in the lungs, was highly variable. Although full recovery of a multiciliated epithelium was observed, the contribution of donor cells versus spontaneous repair or paracrine effects remained unclear.

In recent years, more mature PSC‐derived lung epithelial populations have been transplanted, particularly those with stem cell properties, such as basal cells and AT2 cells. Basal cells derived from human iPSCs and mouse ESCs exhibit long‐term self‐renewal and multilineage differentiation capacity in vivo, forming a tracheal epithelium structurally and compositionally similar to native tissue up to 2 years, contributing to 32% of the recipient tracheal epithelium, and engrafting into intrapulmonary airways 42 days post‐transplantation [76, 80, 81, 142]. Mouse ESC‐derived alveolar epithelial progenitors intratracheally delivered into bleomycin‐injured mice engrafted and matured in the alveolar regions, forming AT1 and AT2 cells [88]. FACS quantification showed that fluorescent donor‐derived cells accounted for 1.4% of all lung epithelial cells. Other studies that transplanted distal airway‐ or alveolar‐fated epithelial cells reported engraftment in the lower regions of the lungs and expression of AT1 and AT2 markers, RAGE, and SPC [143], as well as the ability to delay progression of fibrosis and emphysema [144, 145]. Collectively, these studies confirmed the long‐term retention and function of PSC‐derived lung epithelial cells in vivo, thus encouraging their use as a viable and potentially superior alternative to adult lung progenitors.

Another emerging strategy, known as “interrupted reprogramming,” involves transient expression of iPSC reprogramming to partially dedifferentiate mature lung epithelial cells, such as club and AT2 cells, into a proliferative, progenitor‐like state while acquiring multipotency to generate other epithelial lineages [146, 147]. In vivo, reprogrammed club and AT2 cells have shown therapeutic benefit in models of cystic fibrosis and IPF.

#### 3.2.3. Practical Considerations of Developing Cell Therapies for Respiratory Diseases

**Figure 3:**
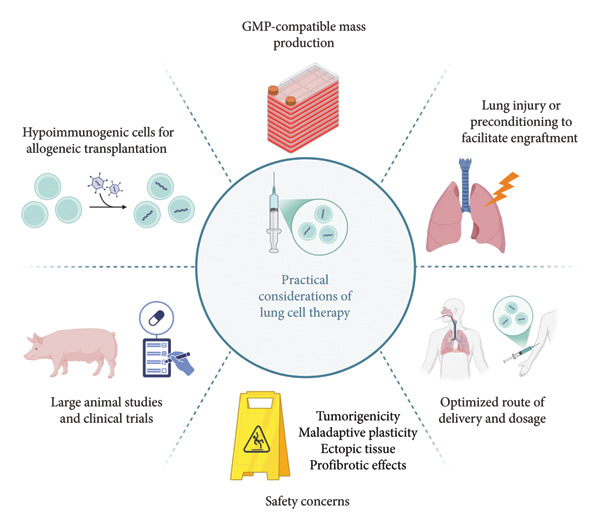
Practical considerations of developing cell therapies for respiratory diseases. Created with Biorender.com.

Several practical barriers must be overcome to develop clinically applicable lung cell therapies, as outlined in Figure 3.

Early studies have highlighted the need for some degree of tissue damage, achieved through disease models or preconditioning regimens, to enable cell engraftment [148]. Before the emergence of clinically relevant models, total body irradiation was commonly used, but stringent control of its dose is required to prevent recipient mortality while ensuring sufficient lung injury [149, 150]. Current models can be broadly classified into two categories: those that mimic respiratory disease, such as influenza infection, bleomycin‐induced pulmonary fibrosis, and hyperoxia, and those, while less physiologically relevant, designed to vacate endogenous niches of the pulmonary epithelium. Examples of the latter include naphthalene preconditioning, which depletes club cells, SO_2_‐induced epithelial injury, and polidocanol‐induced trachea denudation. Polidocanol delivery via a microbronchoscope can further achieve precise and targeted denudation of the intrapulmonary epithelium [142]. One recent study developed a preconditioning regimen potentially suitable for clinical applications by combining low‐dose irradiation with the substitution of naphthalene by cyclophosphamide, a clinically approved reagent for treating various diseases [135].

Total body irradiation can be used to inhibit the recruitment of endogenous BMCs, preventing them from competing with the transplanted cells for injury repair. Administration of a sublethal dose of irradiation following naphthalene preconditioning enhanced the engraftment of donor‐derived cells in recipient lungs [132, 133]. Busulfan is a myelosuppressive drug that can also attenuate BMC recruitment. Preconditioning recipients with a combination of naphthalene and busulfan significantly improved retention of transplanted BMCs [151, 152]. Moving forward, it appears that inducing tissue damage and delaying endogenous repair are both required to maximize cell engraftment.

The route of administration can also be optimized to enhance cell engraftment. Early reports of engraftment of intravenously infused BMC were challenged and attributed to imaging artifacts and cell autofluorescence [4]. Other studies that delivered BMCs intratracheally observed much greater engraftment and long‐term retention [151–153]. Ghosh et al. developed a protocol for intratracheal transplantation of basal cells into naphthalene‐injured NSG mice, which resulted in a 20∼25% reconstitution of airway epithelial cells [154]. The use of synthetic scaffolds that mimic the ultrastructure of the lung parenchyma, such as Gelfoam sponge, gelatin‐based 3D microbubbles, and silk fibroin‐collagen vitrigel membrane, may also promote the retention and maturation of transplanted cells [155–157].

The techniques for mass production of cells for lung cell therapy lag behind other fields of regenerative medicine research. Nevertheless, recent studies have made progress in selectively expanding specific epithelial cell populations in vitro. Dual SMAD inhibition enables long‐term expansion of airway basal cells and maintenance of a multipotent state [79]. An optimized lung progenitor medium is capable of selectively propagating SOX9+ distal lung progenitors over other lung cell types [89]. Directed differentiation protocols have incorporated these findings to generate induced basal cells and AT2 cells from PSCs [80, 88]. These protocols may be scaled up to produce large quantities of therapeutic lung cells. Cell production must also follow Good Manufacturing Practices (GMP). Currently, several GMP‐compliant methods exist to expand ESCs and iPSCs, typically in suspended clusters or feeder‐free human‐derived matrices [158–161]. Although GMP‐compliant stem cell lines have been differentiated into alveolar macrophages [162–164], no such methods exist for generating lung epithelial cells for therapy.

Finally, for the therapeutic benefits to persist, transplanted cells need to be accepted into the host long‐term and evade recognition by the immune system. Autologous transplantation is a possibility, but from a clinical perspective, it is much more efficient to have a source of universal therapeutic cells. Genetic engineering approaches have emerged in recent years to generate hypoimmunogenic and immune‐cloaked cells capable of inducing immune tolerance in allogeneic hosts [165–168]. These strategies may complement each other by targeting different branches of the immune system, ultimately inducing complete tolerance toward transplanted cells.

Key safety concerns must also be addressed. Long‐term tumor risk remains insufficiently studied, with at least one report of teratoma formation, underscoring the need for robust purification of differentiated cells and incorporation of safety switches such as suicide genes. Quality control measures are critical, including spike‐in assays that detect rare undifferentiated PSC colonies, in vivo teratoma formation assays, and molecular detection of PSC‐specific transcripts. Ectopic tissue formation is another major risk. Transplanted cells may display maladaptive plasticity and differentiate into unintended lineages, or migrate away from the delivery site, leading to aberrant nonepithelial masses that disrupt lung function. This may be mitigated through local delivery strategies, transplantation of terminally differentiated cells, and the use of homing or retention cues. Complementary monitoring approaches, including longitudinal imaging, lineage tracing of biopsy samples, and detection of circulating donor‐derived DNA or cells, can ensure early detection of inappropriate cell fates. Transplanted cells or their secreted factors may also inadvertently stimulate resident fibroblasts or macrophages and drive profibrotic programs. In vitro coculture assays with those cell types may be necessary to monitor their activation, coupled with cytokine profiling after transplantation, as well as careful dosing of cells to avoid excessive niche signaling.

There are currently relatively few approved clinical trials for treating chronic lung diseases, and progress on these has been limited. Of 8810 total clinical trials involving stem cell therapies listed on ClinicalTrials.gov, only 468 focused on treating lung injury or disease, with 384 yet to pass Phase II testing, indicating that the majority have not yet been proven to exhibit any therapeutic effect in patients. Most clinical trials employ mesenchymal stem cells to reduce emphysema or inflammation rather than to repair damaged tissue [169]. A few trials have begun to test the repair capabilities of autologous lung stem cells, specifically to treat ILDs, but these trials are not common, and very few have been completed.

## 4. Conclusion and Future Directions

Our understanding of the lung epithelium and the cell populations that can be leveraged for lung repair and regeneration is becoming increasingly comprehensive. Basal cells and AT2 cells remain the predominant stem cell populations in the airway and alveolar epithelium, respectively, playing crucial roles in maintaining epithelial homeostasis and driving regeneration in response to injury. Several facultative progenitor populations residing in specific niches, such as v‐Club cells and BASCs at the BADJ, may contribute to epithelial repair and regeneration in certain disease contexts. The potential roles of rare lung epithelial cells, including ionocytes, PNECs, and tuft cells, also warrant further exploration.

Differentiation protocols to generate these populations have been established and extensively optimized. Although growth factors and culture conditions are relatively well‐defined, biophysical parameters such as geometry and cyclical stretch have received less attention and could be integrated to promote the expansion of specific populations. Mathematical modeling can be used to predict how various factors impact cell fate decisions, with the goal of improving both yield and efficiency. Furthermore, by analyzing omics data from fetal and adult, healthy, and diseased lungs, we may discover additional transitional cell states with regenerative potential, as well as rare or novel cell populations that could be relevant to certain disease conditions.

PSC‐derived respiratory disease models accurately recapitulate key disease characteristics and patient‐specific defects, making them valuable tools for screening drugs tailored to individual patients and advancing personalized medicine. However, many models still fall short of fully mimicking in vivo conditions, particularly with respect to cell–cell and cell–matrix interactions. Integrating lung‐on‐a‐chip technology, which combines lung epithelium with vasculature, may bridge this gap.

The in vivo application of adult and PSC‐derived lung epithelial cells has demonstrated the feasibility of such approaches across various disease contexts, though success has primarily been limited to rodent models. Most studies demonstrated proof of principle for airway and alveolar engraftment, with some showing functional improvements. However, rodent models tend to repair injuries rapidly, can tolerate partial chimerism, and often fail to recapitulate disease characteristics and the immune complexity of the human lung. To address these limitations, future work should increasingly incorporate large animal models that more closely mimic human respiratory disease and immune complexity. Clinically relevant preconditioning strategies, optimized dosing, and delivery regimens all need to be in place to achieve broad and homogeneous epithelial coverage with functional integration. In addition to traditional assessments of histologic engraftment, safety, feasibility, and efficiency, translational studies should incorporate quantitative, disease‐specific endpoints. Examples include airway surface liquid homeostasis and mucociliary clearance for cystic fibrosis, gas exchange efficiency, radiographic disease severity, and lung mechanics such as resistance, compliance, and elastance for pulmonary fibrosis. Equally important is the evaluation of host immune responses to donor cells, as well as the persistence of transplanted cells under repeated injury repair cycles during infections or exposure to environmental insults. These metrics would provide more predictive benchmarks for clinical success.

**Nomenclature**
AFE: Anterior foregut endoderm
ALI: Air–liquid interface
ARDS: Acute respiratory distress syndrome
AT1: Alveolar type 1
AT2: Alveolar type 2
BADJ: Bronchioalveolar duct junction
BASC: Bronchioalveolar stem cell
BMC: Bone marrow–derived cell
BMP: Bone morphogenetic protein
cAMP: Cyclic adenosine monophosphate
CCSP: Club cell secretory protein
COPD: Chronic obstructive pulmonary disease
CPM: Carboxypeptidase M
DE: Definitive endoderm
ECM: Extracellular matrix
ESC: Embryonic stem cell
FGF: Fibroblast growth factor
HPS: Hermansky–Pudlak syndrome
IBMX: Isobutylmethylxanthine
IL: Interleukin
ILD: Interstitial lung disease
IPF: Idiopathic pulmonary fibrosis
KRT: Cytokeratin
LATS: Large tumor suppressor kinase
LNEP: Lineage‐negative epithelial progenitor
NSG: NOD‐scid IL2Rγ^null^
PNEC: Pulmonary neuroendocrine cell
PSC: Pluripotent stem cell
SCA1: Stem cell antigen 1
Scgb: Secretoglobin
scRNA‐seq: Single‐cell RNA sequencing
SHH: Sonic hedgehog
SPC: Surfactant protein C
TGF‐β1: Transforming growth factor β1
TNF‐α: Tumor necrosis factor alpha
TTF‐1/NKX2.1: Thyroid transcription factor 1
v‐Club: Variant club cells

## Disclosure

Golnaz Karoubi, Muyang Zhou, Dana Brinson, and Cindy Lei approved the final version of the manuscript for submission.

## Conflicts of Interest

The authors declare no conflicts of interest.

## Author Contributions

Muyang Zhou, Dana Brinson, and Cindy Lei drafted the manuscript and prepared figures. Golnaz Karoubi, Muyang Zhou, Dana Brinson, and Cindy Lei edited the manuscript.

## Funding

This work was supported by a Cystic Fibrosis Canada Grant (Grant Number 918167) from the Henry White Kinnear Foundation to Golnaz Karoubi.

## Data Availability

Data sharing is not applicable to this article as no datasets were generated or analyzed during the current study.
